# Transcriptional profiling of Arabidopsis root hairs and pollen defines an apical cell growth signature

**DOI:** 10.1186/s12870-014-0197-3

**Published:** 2014-08-01

**Authors:** Jörg D Becker, Seiji Takeda, Filipe Borges, Liam Dolan, José A Feijó

**Affiliations:** 1 Instituto Gulbenkian de Ciência, 2780-156, Oeiras, Portugal; 2 Department of Cell and Developmental Biology, John Innes Centre, Norwich NR4 7UH, UK; 3 Department of Plant Sciences, University of Oxford, South Parks Road, Oxford OX1 3RB, UK; 4 Department of Cell Biology and Molecular Genetics, University of Maryland, 0118 BioScience Research Bldg, College Park 20742-5815, MD, USA; 5 Present address: Cell and Genome Biology, Graduate School of Life and Environmental Sciences, Kyoto Prefectural University, Kitaina-Yazuma Oji 74, Seika-cho, Soraku-gun, Kyoto 619-0244, Japan; 6 Present address: Cold Spring Harbor Laboratory, Cold Spring Harbor 11724, NY, USA

**Keywords:** Pollen, Pollen tube, Root hair, Transcriptome, Apical growth, Tip growth, Apical signature, Arabidopsis

## Abstract

**Background:**

Current views on the control of cell development are anchored on the notion that phenotypes are defined by networks of transcriptional activity. The large amounts of information brought about by transcriptomics should allow the definition of these networks through the analysis of cell-specific transcriptional signatures. Here we test this principle by applying an analogue to comparative anatomy at the cellular level, searching for conserved transcriptional signatures, or conserved small gene-regulatory networks (GRNs) on root hairs (RH) and pollen tubes (PT), two filamentous apical growing cells that are a striking example of conservation of structure and function in plants.

**Results:**

We developed a new method for isolation of growing and mature root hair cells, analysed their transcriptome by microarray analysis, and further compared it with pollen and other single cell transcriptomics data. Principal component analysis shows a statistical relation between the datasets of RHs and PTs which is suggestive of a common transcriptional profile pattern for the apical growing cells in a plant, with overlapping profiles and clear similarities at the level of small GTPases, vesicle-mediated transport and various specific metabolic responses. Furthermore, cis-regulatory element analysis of co-regulated genes between RHs and PTs revealed conserved binding sequences that are likely required for the expression of genes comprising the apical signature. This included a significant occurrence of motifs associated to a defined transcriptional response upon anaerobiosis.

**Conclusions:**

Our results suggest that maintaining apical growth mechanisms synchronized with energy yielding might require a combinatorial network of transcriptional regulation. We propose that this study should constitute the foundation for further genetic and physiological dissection of the mechanisms underlying apical growth of plant cells.

## Background

Current views on the control of cell and organ development are anchored on the notion that phenotypes are defined by precise networks of transcriptional activity, acting in a concerted way through a specific combination of transcription factors to specify cell fate [1]. A direct test of this general principle is facilitated by precise transcriptome analysis using microarrays or RNAseq [2]. This approach in combination with Fluorescence Activated Cell Sorting (FACS), has allowed the characterisation of transcriptomic profiles of isolated cells from simple organs, such as pollen [3]-[5], or more complex ones like roots [6],[7]. The large amounts of information in different databases allow formal analysis of the transcriptional profiles of specific cell types or organs, holding the promise that subsequently these can be distilled into specific transcriptional signatures. At the moment this holy grail of transcriptional regulation is still unattainable, although the majority of these large scale biology approaches end up being extremely useful to the development of smaller scale approaches, focused on a gene or small group of genes [2]. There are likely to be multiple reasons for this limitation, including (1) the limited understanding of additional levels of post-transcriptional/epigenetic regulation that define the final phenotype, (2) the absence of a proper understanding at a formal/mathematical level of network organization and functioning, or (3) these transcriptional profiles do not translate into any sort of accessible mechanistic profile, but are an emergent property of the complexity of other underlying levels of organization based on fundamental chemical and physical properties of DNA and proteins. There is no easy way to circumvent these limitations at our present understanding of biology, but usable clues could arise from applying an analogue to comparative anatomy at the cellular level, such as searching for conserved transcriptional signatures that could be used for further genetic or physiological dissection [8],[9]. Such an approach can be conceptually rooted into evolutionary developmental biology (evo-devo), in which specific and defined small gene-regulatory networks (GRNs) may act as defined modules that may have been co-opted during evolution to perform related functions [10]. Modular GRNs are intrinsically robust and quasi-independent complexes of genes, allowing the possibility of disentangling evolutionary pathways through comparison with similar modules from unrelated species or organs. This architectural feature of the modules, coupled to their power to generate diversity, makes inter-GRN connection elements major targets of adaptive evolution [11]. Plant-microbe interactions have been recently proposed to constitute an attractive system to test some of these concepts, as the communication module seems to have been both phylogenetically re-deployed and functionally adapted along co-evolution of both plants and microbes [12].

Apical growth in filamentous cells is a striking example of conservation of structure and function in plants. As opposed to most plant cells, which grow diffusively over large volumes, these are defined by growing over a relatively small volume at the tip, by exocytosis of specific cell wall precursors [13],[14]. This form of growth is common among fungi and in some animal cells (neurite outgrowth during the development of the nervous system; see [15]), and in flowering plants it occurs only in root hairs and pollen tubes. Despite differences, growth and morphogenesis is similar in these two cell types [16]-[18] and as they are functionally skewed towards the same objective: perceive the surrounding environment and process this information to direct growth. Previous studies suggested that the molecular and physiological mechanisms employed to direct growth are likely conserved between pollen tubes and root hairs [19],[20]. This conservation is especially well observed at the level of the cytoskeleton organization, membrane trafficking and endo/exocytosis and signalling pathways mediated by calcium, phosphoinositide, ROPs and ROS [18],[20]-[24]. Developmental definition by specific transcription factors is well described for root hairs (see for example [25],[26]) and pollen grains [27],[28]. Previous transcriptional profiling of pollen and sperm [3],[4] allowed the search of conserved GRNs that exist in the two different cell types that compose the male gametophyte. In comparison, root hairs must be seen in the context of the root, a very complex organ where various hierarchical levels of transcriptional integration are expected [7]. While much is known about root transcriptomics in general, the profile of isolated root hairs is still lacking, limiting the possibility of comparative analysis with pollen tubes, and search for conserved transcriptional network motifs. The advent of more powerful and revealing ways of imaging signal integration in roots (see for example [29],[30]) makes it even more obvious the need of specific transcriptomics of root hairs, one of the physiologically more important cell types in roots.

Here we compare the transcriptional profile of isolated root hairs and pollen with other cell and organ types to test the hypothesis that there are conserved transcriptomic signatures that define functions in similarly growing cells. Root hair transcriptomics was previously approached by a number of studies using FACS of labelled root cell types and nuclei, respectively [6],[7],[31]-[33], by dataset subtraction from root hair development mutants [34],[35], or by a combination of mutants and FACS [36]. Here we developed a new way of isolating mRNA directly from mechanically purified frozen wild type root hairs. We conclude that root hairs and pollen have highly overlapping transcriptional profiles, with clear similarities at the level of small GTPases, vesicle-mediated transport and various specific metabolic responses, likely defining the unique regulatory processes that occur in these cell types. We propose that this study should constitute the foundation for further genetic and physiological dissection of the mechanisms underlying apical growth of plant cells.

## Results

### Isolation of *Arabidopsis* root hairs

**Figure 1:**
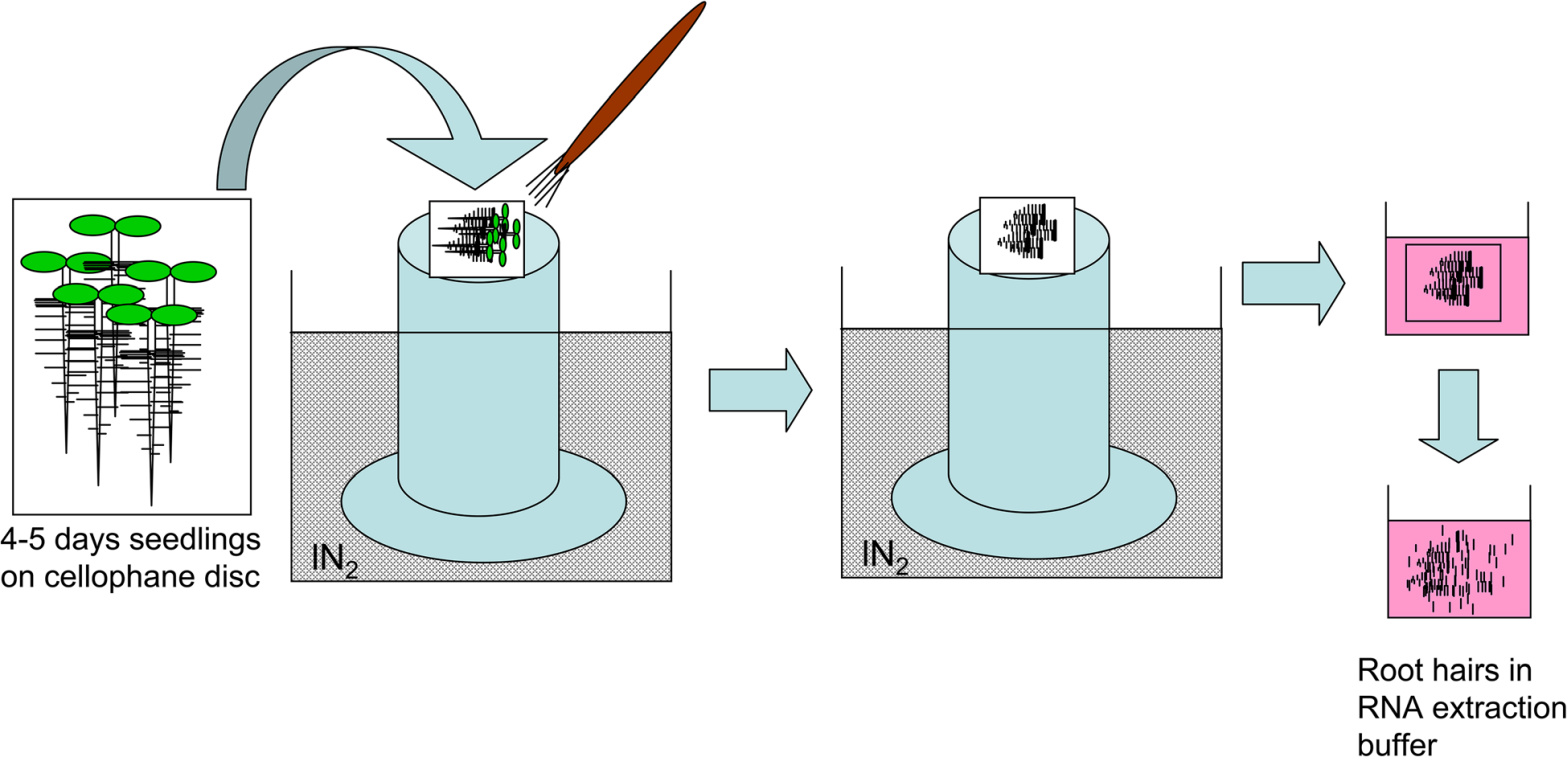
**Schematic workflow of root hair isolation.***Arabidopsis* Col-0 plants were grown on cellophane disc for 4 or 5 days. The cellophane discs on which plants grew were transferred on the top of an aluminium tower placed in liquid nitrogen, left for 1-2 seconds, and plants except for root hairs were removed by brush. Root hairs attached on the cellophane disc were released in RNA extraction buffer. Other tissues such as root tips in the buffer were removed carefully with forceps under a stereomicroscope.

**Figure 2:**
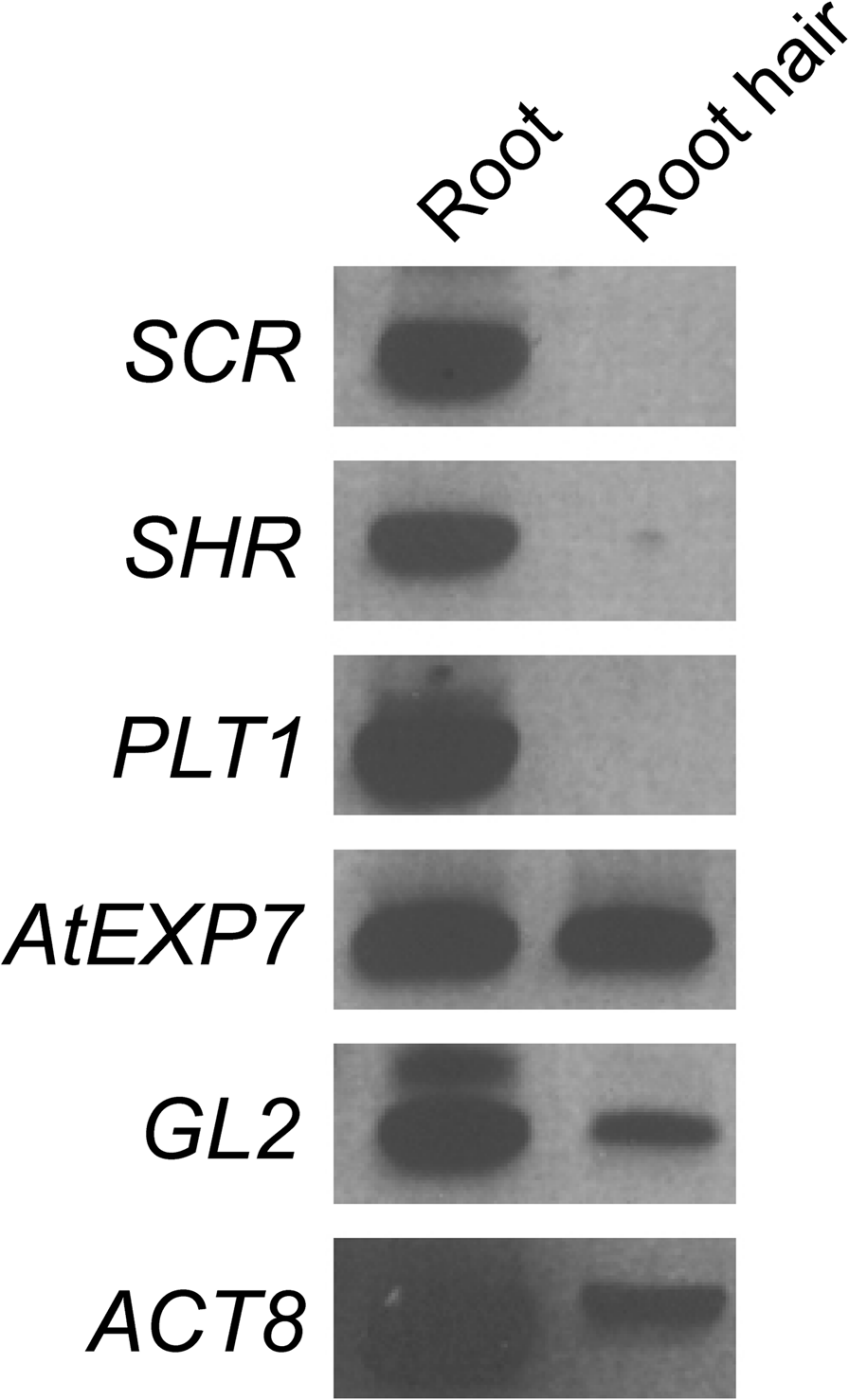
**RT-PCR of root and root hair RNA, respectively.** Results from negative controls using *SCR*, *SHR* and PLT1 show no contamination from inner cell layers in roots. *AtEXP7* and *ACT8* expression confirm the root hair RNA in the sample. *GL2*, which is preferentially expressed in atrichoblast but also expressed in low levels in some trichoblast, was also detected in root hair RNA.

The purity of total RNA isolated from root hairs was important for this study, because the slightest contamination would have obscured a potential apical growth signature. Therefore, we established a method using an aluminum tower partially immersed in liquid nitrogen and a brush to isolate root hairs from Arabidopsis seedlings (Figure 1, see Methods). To determine the quality of the total RNA isolated from root hairs, several genes expressed in specific cell types in roots were investigated by RT-PCR (Figure 2). *SCARECROW* (*SCR*) expressed in cortex, *SHORT ROOT* (*SHR*) in stele, and *PLETHORA1* (*PLT1*) in stem cells, were amplified from root cDNA but not from root hair cDNA [37]-[39], whereas *Arabidopsis thaliana EXPANSIN7* (*AtEXP7*), which has been shown to be expressed in root hair cell files [40], was detected both in root and root hair cDNA. *ACTIN8* (*ACT8*), expressed throughout the plant including the root hairs [41] was used as a positive control. *GLABRA2* (*GL2*) is preferentially expressed in non-hair cells of the root epidermis but is also expressed in low levels in some root hair cells [42],[43], and was detected in our root hair sample. Moreover, *ENHANCER OF TRY AND CPC1* (*ETC1*) and *MYB23*, both of which are non-hair cell markers [44],[45], were called “absent” in our microarray data. Together, our data indicated that the extracted RNA was rich in root hair specific transcripts.

### Root hairs and pollen overlap significantly in their transcriptional programs

We obtained the transcriptional profile of the root hairs using Affymetrix Arabidopsis ATH1 arrays. 11,696 genes were detected as expressed, corresponding to 51% of the transcripts represented on the array (mean percentage of Present calls). The expression profile of root hairs was compared with those of cell sorted hydrated pollen grains (29% of Present calls), leaves (62%), seedlings (68%), siliques (69%), flowers (68%) [5] as well as ovules (67%) and unpollinated pistils (69%) [46]. In addition, we reanalyzed expression data of single cell types of roots [6],[47] resulting in 58% of Present calls for stele, 62% for endodermis plus quiescent center, 66% for cortex and 53% for epidermal atrichoblasts. Thus, the number of genes expressed in root hairs is significantly higher than in pollen, but smaller than in other vegetative tissues and even in a number of root cell types. It is however similar in root hairs and epidermal atrichoblasts.

**Figure 3:**
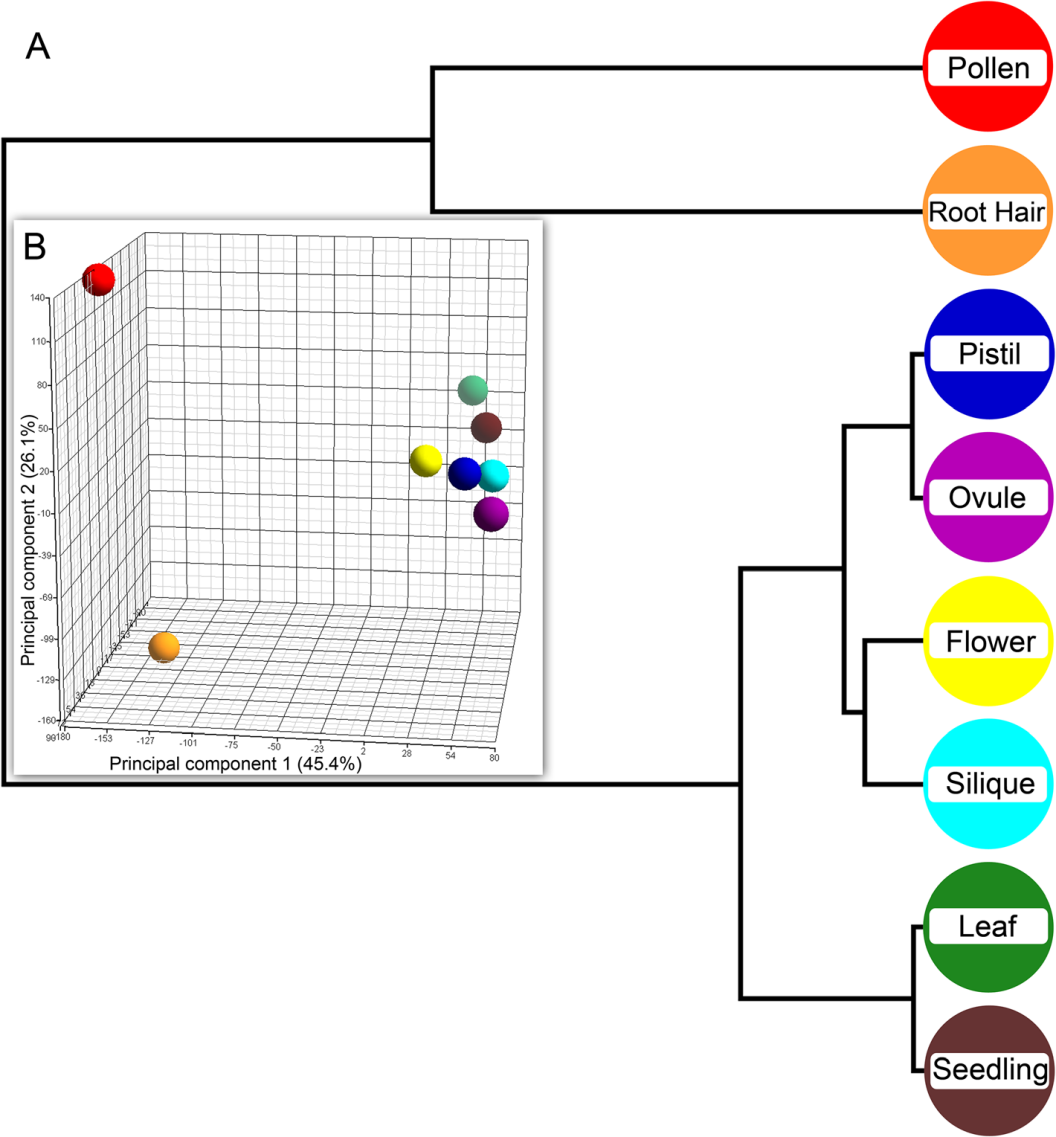
**Principal component analysis and hierarchical clustering of Arabidopsis transcriptome data.(A)** Principal component analysis is an exploratory technique used to describe the structure of high dimensional data, e.g. derived from microarrays, by reducing its dimensionality. Here, expression values for 22.800 genes in 8 tissue/cell types are projected onto the first three principal components. The first principal component separates pollen and root hairs from the other tissues, while the second and third principal components show a further, though less significant, separation of the samples. **(B)** Hierarchical clustering is used to group similar objects into “clusters”, producing a tree (called dendrogram) that shows the hierarchy of the clusters. The dendrogram shows a clear separation of a pollen and root hair cluster from a cluster including the other sample types.

When the expression data derived from our data sets is subjected to principal component analysis and hierarchical clustering, closely related or overlapping tissues like seedling and leaves, pistils and ovules and siliques and flowers form sub-clusters (Figure 3A). Interestingly root hairs form a sub-cluster with pollen and not with any of the tissues. Principal component analysis shows a similar picture with root hairs and pollen being clearly separated from the other tissues in the first principal component (Figure 3B). Cell types with apical growth type (root hairs and pollen) are conclusively separated from tissues containing cells only with diffuse growth type (pistils, ovules, siliques and leaves) or even a mixture of diffuse and apical growth cell types as found in flowers containing pollen and seedlings containing root hairs. This result statistically shows a relation between the datasets which is suggestive of a common transcriptional profile pattern for the apical growing cells in a plant. Importantly, other root cell types [33] do not cluster together with pollen and root hair samples (Additional file 1: Figure S1 and Additional file 2: Table S1 for PCA loadings). This is an indication that the separation observed is not solely based on green versus non-green tissue features, although one has to keep in mind that comparison with the root cell type datasets might be confounded by protoplasting and FACS effects. 1814 genes show enriched expression in root hairs in relation to expression levels in leaf, pistil, ovule and silique samples. When compared with “root hair genes” as defined in other studies [7],[31],[32],[34],[36] the highest overlap (125 genes out of 153) is achieved with the “core set hair genes” identified by Bruex et al. [36] (Additional file 3: Table S2).

### Analysis of pollen tube and root hair transcriptomes reveals an apical growth signature

**Figure 4:**
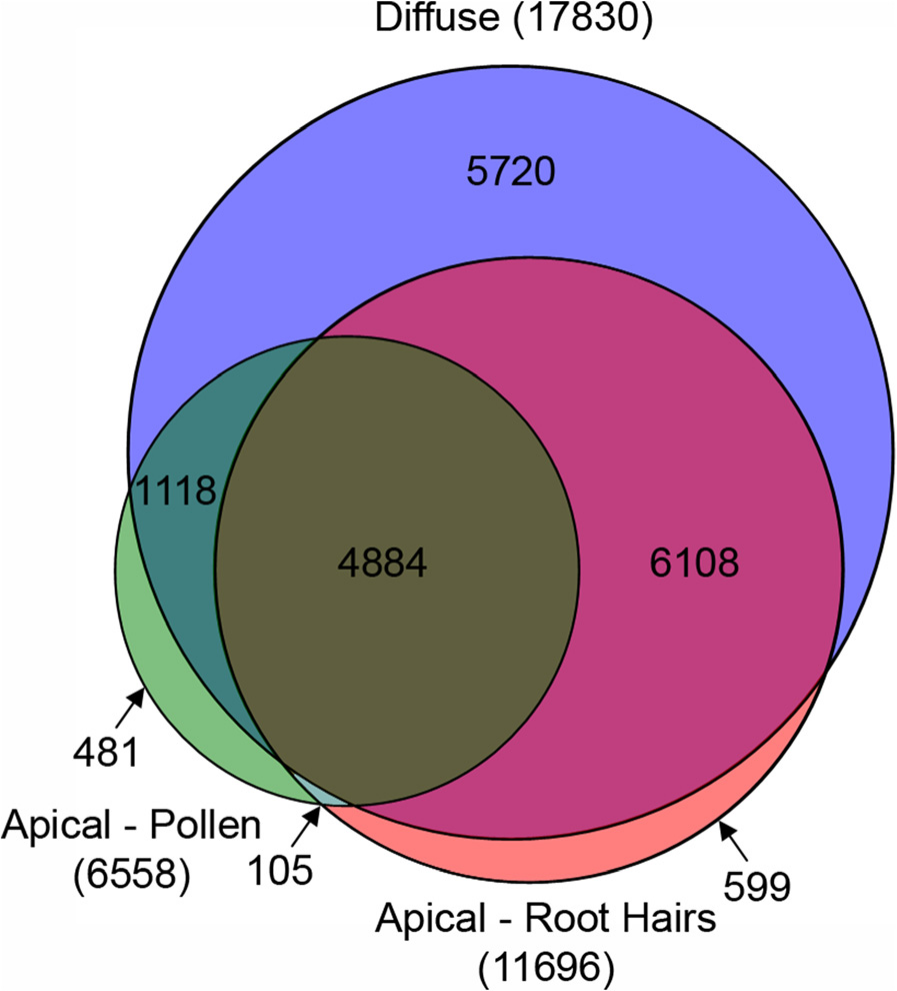
**Venn Diagram depicting the number of expressed genes (as defined by Present calls) in apical growing and diffuse cell types, and their respective overlaps.** Flowers and seedlings were excluded from this analysis, since they contain pollen and root hairs, respectively.

We hypothesized that the differences observed in the transcriptional profiles would predominantly derive from transcripts that show enriched or selective expression in root hairs and pollen when compared with tissues containing solely cells with diffuse growth type. Of the 4989 genes expressed in both pollen and root hairs our comparative analysis identified 277 genes as showing enriched expression in these apical growing cells (Additional file 4: Table S3). Based on comparison with our restricted data set of 4 tissues with cell types showing diffuse growth, 105 genes are selectively expressed in apical growing cells (Figure 4). However, extending this comparison by including other Arabidopsis tissue types and developmental stages (Schmid et al. 2005) strictly containing only cell types with diffuse growth type, reduces this list of selectively expressed genes to 49 (Table 1). Transcriptome analysis of growing pollen tubes of Arabidopsis has shown that there is a moderate increase in transcript diversity and abundance when comparing growing pollen tubes with hydrated pollen grains [48]. To assess if we are missing potential apical growth signature genes we crossed our list of 1814 root hair enriched transcripts with the list of genes up-regulated during pollen tube growth [48] and our 4989 genes common to mature hydrated pollen and root hairs (Additional file 3: Table S2). 34 of the 41 genes identified as being enriched in root hairs, up-regulated in growing pollen tubes and not in our apical growth list were called Absent in our pollen data and would thus potentially have to be added to our list of 277 apical growth enriched genes, if not being expressed at higher levels in the sporophytic tissues analyzed. Furthermore, in a recent study 104 genes were identified as potential polar cell expansion genes by crossing tobacco pollen tube with Arabidopsis trichoblast transcriptomic data [49]. We found 48 of those genes to be expressed in Arabidopsis pollen and root hairs, three showing enriched expression and none being selective (Additional file 4: Table S3).

**Table 1:** Selectively expressed genes in apical growing cells

**Function**	**Probe set**	**AGI ID**	**Gene**	**Pollen**	**Root hair**	**Enriched**	**FC**	**Pollen FC**	**Root hair FC**
Protein modifications	264284_at	At1g61860	Protein kinase, putative	13390	360	X	72.4	141.2	3.5
Protein modifications	249950_at	At5g18910	Protein kinase family protein	8337	261	X	56.5	110.0	3.0
Protein modifications	251433_at	At3g59830	Ankyrin protein kinase, putative	2322	279	X	19.1	34.2	3.9
Protein modifications	258832_at	At3g07070	Protein kinase family protein	795	2708	X	19.0	8.3	29.7
Protein modifications	267582_at	At2g41970	Protein kinase, putative	2680	2854	X	12.5	12.2	12.8
Protein modifications	263378_at	At2g40180	Protein phosphatase 2C, putative/PP2C, putative	1481	197	X	10.8	19.2	2.4
Protein modifications	248909_at	At5g45810	CIPK19	2006	560	X	6.4	10.2	2.6
Protein modifications	265178_at	At1g23540	Protein kinase family protein	620	133	X	6.3	10.6	2.0
Protein modifications	264127_at	At1g79250	Protein kinase, putative	347	452	X	4.3	3.8	4.7
Protein modifications	253718_at	At4g29450	Leucine-rich repeat protein kinase, putative	233	176			2.9	
Calcium signalling	245036_at	At2g26410	IQD4 (IQ-domain 4); calmodulin binding	12177	1885	X	59.5	105.5	13.6
Calcium signalling	259064_at	At3g07490	AGD11 (ARF-GAP DOMAIN 11)	4146	1254	X	17.5	27.3	7.6
Calcium signalling	254774_at	At4g13440	Calcium-binding EF hand family protein	90	173				1.8
G-protein signalling	259836_at	At1g52240	ATROPGEF11/ROPGEF11 (KINASE PARTNER PROTEIN-LIKE)	3003	282	X	51.8	95.5	8.1
G-protein signalling	260161_at	At1g79860	ATROPGEF12/MEE64/ROPGEF12 (KINASE PARTNER PROTEIN-LIKE)	3436	661	X	44.0	75.0	13.1
G-protein signalling	263458_at	At2g22290	AtRABH1d (Arabidopsis Rab GTPase homolog H1d)	458	674	X	12.2	9.4	14.9
G-protein signalling	254173_at	At4g24580	Pleckstrin homology (PH) domain-containing protein-related / RhoGAP domain-containing protein	782	164	X	4.8	8.0	1.6
G-protein signalling	266190_at	At2g38840	Guanylate-binding family protein	1383	1343	X	2.3	2.5	2.2
Cell wall proteins	263453_at	At2g22180	Hydroxyproline-rich glycoprotein family protein	5725	233	X	37.8	73.0	2.7
Cell wall Proteins	249375_at	At5g40730	AGP24 (ARABINOGALACTAN PROTEIN 24)	19904	13284	X	16.3	19.7	12.9
Cell wall proteins	259720_at	At1g61080	Proline-rich family protein	533	482	X	11.0	11.6	10.3
Cell wall proteins	246872_at	At5g26080	Proline-rich family protein	128	152	X	1.5	1.4	1.6
Cell wall proteins	245159_at	At2g33100	ATCSLD1 (Cellulose synthase-like D1)	11021	55			132.6	
Cell wall proteins	250801_at	At5g04960	pectinesterase family protein	67	8360				114.1
Cell wall proteins	251842_at	At3g54580	Proline-rich extensin-like family protein	447	150			4.2	
Cell wall proteins	265275_at	At2g28440	Proline-rich family protein	488	136			6.2	
Transcription	261643_at	At1g27720	Transcription initiation factor	540	196	X	3.1	4.7	1.6
ENTH	247941_at	At5g57200	Epsin N-terminal homology (ENTH) domain-containing protein / clathrin assembly protein-related	717	108	X	9.3	16.3	2.3
P- and V-ATPases	251405_at	At3g60330	AHA7 (ARABIDOPSIS H(+)-ATPASE 7)	654	2829	X	22.7	8.2	37.2
Lipid degradation	267439_at	At2g19060	GDSL-motif lipase/hydrolase family protein	181	396	X	3.4	2.6	4.2
Vesicle transport	259338_at	At3g03800	SYP131 (syntaxin 131)	13646	603	X	45.0	86.6	3.3
Exocyst	250204_at	At5g13990	ATEXO70C2 (exocyst subunit EXO70 family protein C2)	1428	695	X	16.4	21.1	11.7
Exocyst	245979_at	At5g13150	ATEXO70C1 (exocyst subunit EXO70 family protein C1)	3238	1780	X	15.8	20.5	11.1
Aging	249868_at	At5g23030	TET12 (TETRASPANIN12)	97	1167				19.5
Cytoskeleton organisation	266697_at	At2g19770	PRF5 (PROFILIN5)	5226	165	X	30.4	59.1	1.8
Ion transport	251053_at	At5g01490	CAX4 (cation exchanger 4)	495	342			1.8	
Isoprenoid biosynthesis	257274_at	At3g14510	Geranylgeranyl pyrophosphate synthase, putative	58	112				1.8
Microtubule-based movement	254205_at	At4g24170	Kinesin motor family protein	1216	143	X	13.0	23.5	2.5
Protein Folding	260478_at	At1g11040	DNAJ chaperone C-terminal domain-containing protein	1136	112			17.5	
Pyrophosphatase activity	266765_at	At2g46860	Inorganic pyrophosphatase, putative (soluble)	1024	598	X	10.8	14.2	7.4
Unknown	246592_at	At5g14890	NHL repeat-containing protein	7155	144	X	48.7	95.6	1.8
Unknown	260320_at	At1g63930	Similar to unknown protein (TAIR:AT4G23530.1)	1169	1495	X	18.0	15.8	20.1
Unknown	267051_at	At2g38500	Similar to DTA4 (DOWNSTREAM TARGET OF AGL15-4) (TAIR:AT1G79760.1)	4282	546	X	13.6	24.3	2.8
Unknown	256506_at	At1g75160	Similar to unknown protein (TAIR:AT5G05840.1)	958	310	X	12.8	19.9	5.6
Unknown	266674_at	At2g29620	Similar to unknown protein (TAIR:AT1G07330.1)	337	839	X	9.7	5.6	13.8
Unknown	251135_at	At5g01280	Similar to proline-rich family protein (TAIR:AT3G09000.1)	134	1001	X	7.8	1.6	14.0
Unknown	249185_at	At5g43030	DC1 domain-containing protein	346	487	X	4.8	4.0	5.6
Unknown	251047_at	At5g02390	similar to unknown protein (TAIR:AT1G07620.1)	388	321	X	4.6	5.2	4.0
Unknown	252987_at	At4g38390	Similar to unknown protein (TAIR:AT1G76270.1)	106	423				5.5
Unknown	254972_at	At4g10440	Dehydration-responsive family protein	135	144				
Unknown	260195_at	At1g67540	Unknown protein	540	51			6.5	

The first column shows the functional classification of the gene (see also Figure 7). The second column depicts the Affymetrix probe set, followed by TAIR locus (AGI ID) assigned to this probe set and gene annotation in the third and fourth column. In columns five and six the expression values for pollen and root hairs, respectively, are given. The following three columns (7 to 8) depict, if a gene is selectively and /or enriched expressed in root hairs and pollen, followed by the average of the lower confidence bound of the fold change (FC) for apical growing cells. The last two columns give the average FC of pollen and root hairs, respectively.

**Figure 5:**
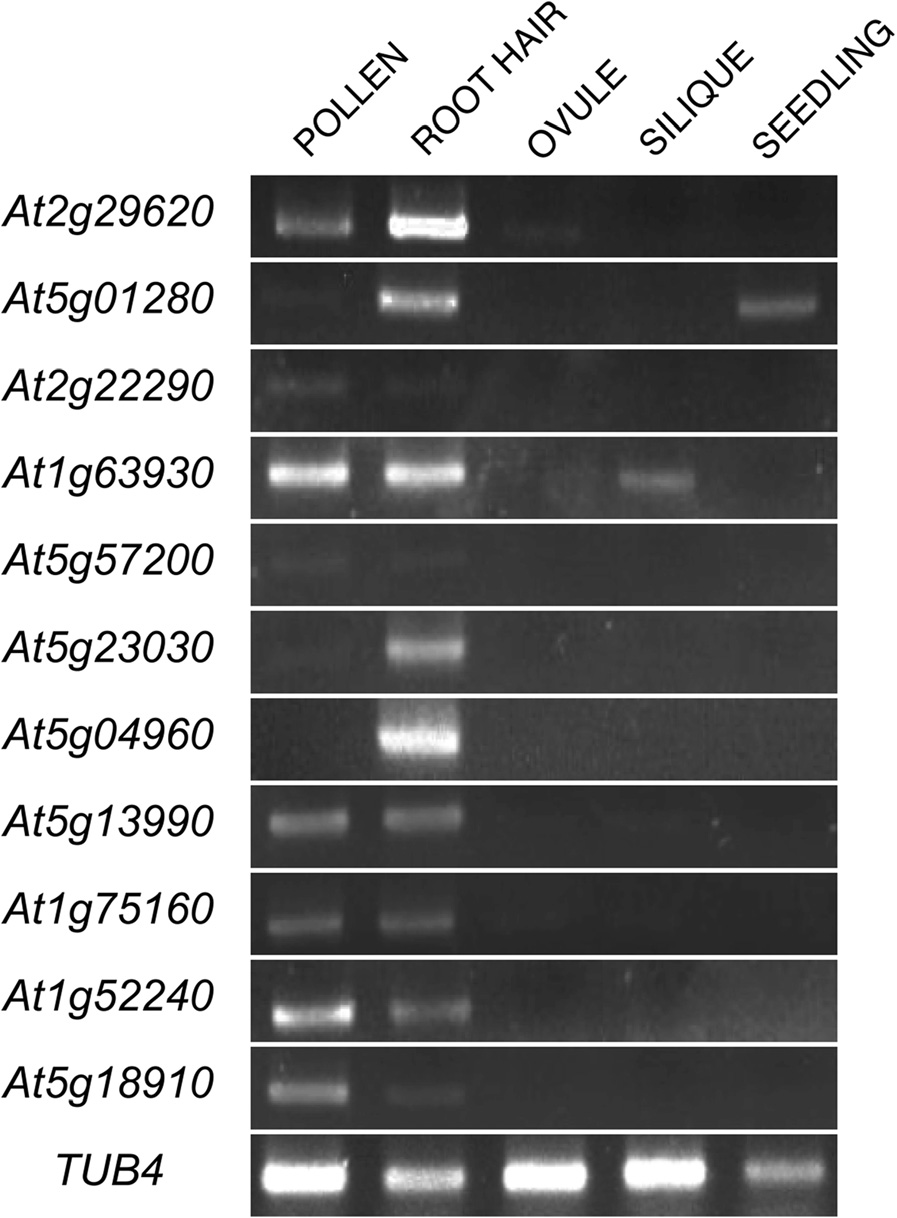
**RT-PCR analysis.** Gel figures for ten genes whose expression was detected only in pollen and root hair samples but not in vegetative tissues (ovule, silique and seedling) by microarray. *TUB4* - tubulin β-4 chain (*At5g04180*) was used as positive control.

To validate our microarray results, we performed RT-PCR analysis for eleven of these apical growth selective transcripts. Ten were detected in both pollen and root hair samples, while *At5g04960* could not be amplified from our pollen cDNA sample (Figure 5), possibly reflecting its low signal value of 67 on the pollen arrays. RT-PCR analyses have additionally shown that even if a transcript is called Absent on a Genechip experiment, it might still be detected by RT-PCR. This holds true for *At2g29620*, *At5g01280* and *At1g63930*, which were detected also in ovules, seedlings and siliques, respectively (Figure 5), although the latter two are likely to be root hair- and pollen-derived, respectively. Thus it seems that ten out of eleven apical growth genes are mainly expressed in root hairs and pollen, which is a significantly positive result to allow downstream analyses based on the array data. In addition, comparing detection levels for pollen and root hair samples confirms a significant correlation between microarray data and the semi quantitative RT-PCR performed.

**Figure 6:**
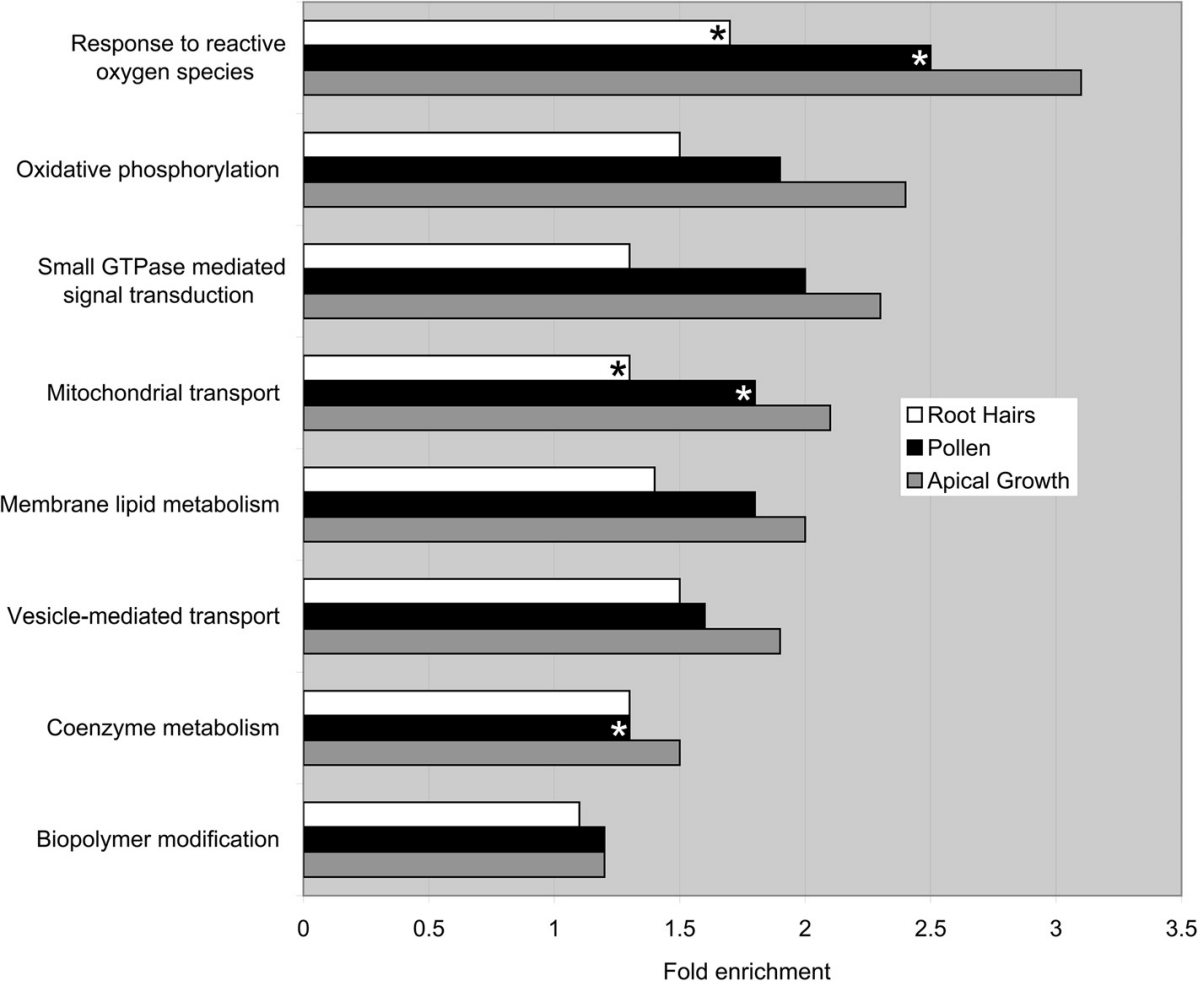
**Functional enrichment analysis of genes expressed in root hairs, in pollen and in both (apical growth) based on Gene Ontology biological process terms.** An asterisk denotes classes that are not statistically significantly enriched in the particular cell type. See Additional file 5: Table S4 for a list of the genes comprising the classes in apical growth.

Next we asked if genes expressed in both pollen and root hairs are functionally skewed towards biological process classes known or expected to be involved in apical cell growth. Our comparative Gene Ontology analysis showed that genes involved in membrane lipid metabolism and vesicle-mediated transport are over-represented in apical growing cells (Figure 6 and Additional file 5: Table S4). In addition energy metabolism, represented by the classes oxidative phosphorylation, mitochondrial transport and coenzyme metabolism, as well as signal transduction, comprising the classes response to reactive oxygen species, small GTPase signaling and biopolymer modification, are over-represented functions in these cell types. Most but not all of these classes are statistically significantly enriched even when the complete set of genes in the root hair and pollen transcriptome, respectively, are analyzed separately (Figure 6).

**Figure 7:**
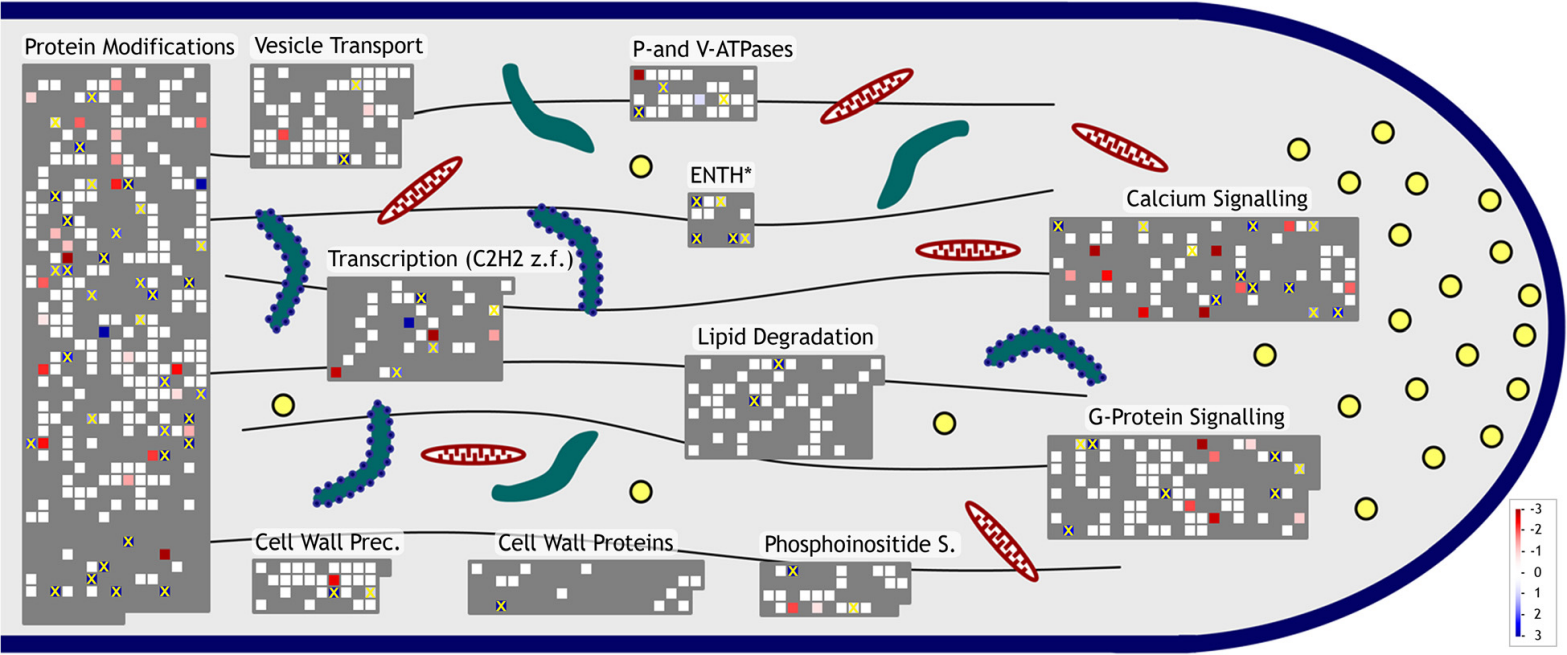
**Gene family analysis of apical versus diffuse growing cell types.** Gene expression data from root hairs and pollen relative to siliques, pistils, ovules and leaves are shown on a scheme depicting shank and tip of an apical growing cell. Genes are symbolized by color-encoded squares (red, down-regulation; blue, up-regulation; white, present call in root hairs and pollen, but no concordant change; grey, Absent call in pollen and/or root hairs; X, selective expression in root hairs and pollen). Abbreviation: ENTH, Epsin N-Terminal Homology domain-containing protein; Prec., Precursor; z.f., zinc finger; S., Signalling.

The MapMan tool [50] was used to map differential gene expression in apical versus diffuse growing cell types on the most relevant gene families (Figure 7 and Additional file 6: Table S5). This detailed gene family and pathway analysis facilitates the identification of primary targets for reverse genetics confirmation of a possible role for respective gene products in apical cell growth.

### Promoters of genes that define the apical growth signature share common cis-elements

**Figure 8:**
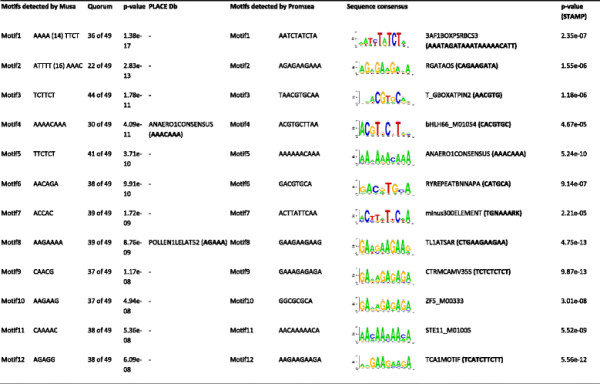
**Motifs reported by MUSA[**[51]**]and Promzea[**[52]**]for 49 promoter sequences of apical growth selective genes.** Motifs detected by MUSA are ranked by p-value, highlighting correspondence to *cis*-elements summarized in PLACE database [53]. The quorum value shows the number of query sequences in which a certain motif stands. The sequence consensus for each motif detected by Promzea was compared to known plant promoter database by STAMP [54], and the results were ranked by p-value. Only the most significant result is shown.

The identification of conserved *cis*-regulatory elements is important to understand regulatory networks and combinatorial gene expression. To identify conserved motifs associated with the apical growth gene expression signature, we analysed the promoter regions of apical growth selective genes. In order to overcome recognized limitations of most motif discovery tools available, from which different motifs are obtained after each run, we performed promoter sequence analysis using two different tools, and compared the results based on sequence consensus alignment and annotation to different plant promoter databases. As expected, different motifs were detected by Musa [51] and Promzea [52] as overrepresented in the promoters of apical growth genes (Figure 8). While we were not able to find correspondence to many of the motifs identified by Musa within the publicly available plant promoter database PLACE [53], it was possible to identify the most statistically significant consensus sequences detected by Promzea using STAMP [54]. We found common elements such as the TATA box and pyrimidine patch (Y Patch) elements [55]-[57] that generally appear near the transcriptional start site (TSS). This might be the case for the TCTTCT and TTCTCT motifs (Figure 8), which probably form part of the higher plant-specific core promoter element Y Patch. Musa was able to detect the AGAAA motif, which is a *cis*-regulatory element of the *Lat52* promoter that is preferentially active in the vegetative cell during pollen maturation [58].

Interestingly, the only motif detected by both tools was A*AAACAAA,* a *cis*-element that was previously detected in the promoters of genes whose expression is induced anaerobically [59]. It is likely that both pollen tube and root hairs growth might sometimes suffer hypoxia, owing to submergence either inside sporophyte tissues or by water flooding, respectively. In fact, an alternative to mitochondrial respiration has been previously characterized in species with bicelullar pollen such as tobacco and petunia [60]-[63]. Oxygen availability was never a limiting factor for pollen germination in vitro, while ethanol fermentation either involving alcohol dehydrogenase (ADH) and pyruvate carboxylase (PDC) pathways were demonstrated to be essential for pollen tube growth and fertilization. Taken together, our results suggest that maintaining apical growth mechanisms synchronized with energy yielding might require a combinatorial network of transcriptional regulation.

## Discussion

Cell growth takes place at a restricted area at the cell apex in pollen tubes and root hairs, a process called tip or apical growth [13],[14]. While many components of the mechanism required for growth of these extremely polarised cells also occur in other cell types that grow by diffuse growth, our analysis of the root hair and pollen transcriptome demonstrates that tip growing cells are defined by a common set of proteins that carry out activities required for tip-growth. We propose that the core set of genes that comprise this apical signature encode proteins that are active in a variety of cellular activities that are required for this mode of cell elongation.

As part of this study we have developed a novel method to isolate growing and mature root hairs directly from seedlings. It circumvents problems associated with methods used in other studies aiming at identifying root hair-rich expression, e.g. by relying on mutants with decreased or increased abundance of root hairs [34]-[36] or on FACS sorted cells or nuclei [6],[7],[31]-[33],[36]. Altered transcriptional profiles due to the mutations or due to the extensive manipulations needed before FACS in combination with the limitation in purity for the FACS approaches might explain the limited overlap of our root hair enriched gene list with comparable lists from these studies. Further confounding factors are technical differences like the platforms used (RNAseq or different microarrays) and the tissue types used to identify enriched or selective expression. Given these restrictions the 82% overlap with the 153 “core set hair genes” identified by Bruex et al. [36] is remarkable and validates our approach.

It is long known that the growth in both pollen tubes and root hairs is accompanied by similar physiological processes (reviewed by [20]). Probably the best characterised is the formation of a tip-high gradient of cytoplasmic calcium in both cell types and that is required for growth (reviewed by [17],[64]). This local elevation in cytoplasmic calcium concentration is believed to be formed as a result of the activity of channels that transport calcium ions from the outside of the cell to the cytoplasm in the apical region of the cell [65]. It is likely that other physiological processes that are specific to tip growing cells exist and remain to be identified. Our analysis of the pollen and root hair transcriptome has identified sets of genes that are common to elongating pollen tubes and root hairs and may thus define such a suite of apical growth-specific processes. This increases significantly a previously defined list of 104 potential polar cell expansion genes [49]. The genes we have identified encode proteins active in a variety of processes, including signalling, cell wall modification, oxidative phosphorylation, mitochondrial transport and coenzyme metabolism. We therefore propose that the apical-growth gene expression signature defines a suite of cellular activities that, like the tip high calcium gradient, are required for the extension of tip growing cells.

Among the processes that are defined by the apical transcriptome are genes involved in signalling processes that control growth. GTPases are key regulators of signalling cascades in cells that play important roles in the co-ordination of cellular activities during growth (reviewed in [66],[67]). The Rab GTPase homolog H1d (At2g22290) for example is a selectively expressed component of our apical growth signature and has been identified by Lan *et al*. [32] as potential key component of a Rho-signaling network in root-hair differentiation. Reactive oxygen species play important roles in signaling and cell wall modification during growth of pollen tubes and root hairs and genes that are induced in response to reactive oxygen species are components of the apical-growth signature [68]-[70]; reviewed in [17]. It is likely that they are active in aspects of ROS-regulated apical growth in these cell types [71]. We propose that these different sets of signalling modules are central components of the apical growth mechanism.

The coordinated expression of genes in pollen tubes and root hairs likely involves a common set of regulatory elements. *Cis*-regulatory elements in the DNA sequence surrounding a gene play important roles in the control of gene expression. Different *cis*-regulatory elements are required for the induction of gene expression in different cell types or in response to changes in environmental conditions. For example short WHHDTGNNN(N)KCACGWH elements occur in the promoters of genes that are expressed in the root hair of Arabidopsis [35]. Our analysis demonstrates that there are conserved *cis*-regulatory elements in the promoters of genes that are expressed in pollen tubes and root hairs. We found the A*AAACAAA cis*-regulatory element that is found in genes whose transcription is induced in anaerobic conditions. This is consistent with the hypothesis that tip growing cells suffer anoxia, an hypothesis long set forth for pollen tubes [72], and known to have specific adaptions in root hairs [73]. These conserved *cis*-regulatory elements are likely required for the expression of genes of the apical signature, but given the divergent results of the two prediction tools experimental validation will be needed.

## Conclusions

Together our analyses of the pollen tube and root hair transcriptome indicate that there is a core of 277 genes whose expression is higher in these cell types when compared to others in the plant. We propose that the proteins that are encoded by these genes define activities that are common to both cell types. We predict that like the tip-high calcium gradient and the apical production of reactive oxygen species that are required for growth in these cells, these activities will define cellular processes that are required for the growth of tip-growing cells in land plants. Given that the tip-high calcium gradient also occurs in other organisms such as fungi (see for example [74]), future research will define if the processes regulated by genes of the apical signature are active in other tip growing cells of eukaryotes.

## Methods

### Plant growth conditions

Seeds for root hair isolation were sterilized in 5% sodium hypochlorite, washed by water and sown on half strength Murashige and Skoog (Duchefa, Haarlem, The Netherlands) medium (pH 5.8) containing 1% sucrose and 0.8% phytagel.

### Root hair RNA isolation and RT-PCR

The scheme of isolating root hairs is shown in Figure 1. Four to five surface-sterilized seeds of *Arabidopsis thaliana* Columbia (Col-0) were sowed on a 3 cm-diameter cellophane disc of type 325P (AA packaging Ltd, Preston, UK), placed on growth media and incubated horizontally under continuous light for 4 to 5 days. The discs on which plants grew were frozen for 1-2 seconds on an aluminium tower (20 cm height) half-sunk in liquid nitrogen (Figure 1). A small flat paint brush was used to carefully remove the leaves, hypocotyls and roots from the frozen plant tissue, except for root hairs that were retained on the discs. These hairs were collected in RNA extraction buffer. Contaminating root tips were removed under a stereomicroscope.

Total RNA from root hairs was isolated by RNeasy Mini extraction kit (Qiagen, Hilden, Germany) and integrity was confirmed using an Agilent 2100 Bioanalyzer with a RNA 6000 Nano Assay (Agilent Technologies, Palo Alto, CA). Total RNA was reverse-transcribed by Superscript II reverse transcriptase (Invitrogen, Paisley, UK) and used for RT-PCR.

For confirmation of selective expression of apical growth genes we used cRNA amplified from pollen, root hair, ovule, silique and seedling samples to prepare double-stranded cDNA. Five nanograms of each template cDNA were subsequently used in reactions of 35 PCR cycles. The primer sequences for all RT-PCRs are shown in Additional file 7: Table S6.

### Target synthesis and hybridization to Affymetrix GeneChips

The GeneChip experiment was performed with biological duplicates. Root hair total RNA was processed for use on Affymetrix (Santa Clara, CA, USA) Arabidopsis ATH1 genome arrays, according to the manufacturer’s Two-Cycle Target Labeling Assay. Briefly, 100 ng of total RNA containing spiked in Poly-A RNA controls (GeneChip Expression GeneChip Eukaryotic Poly-A RNA Control Kit; Affymetrix) was used in a reverse transcription reaction (Two-Cycle DNA synthesis kit; Affymetrix) to generate first-strand cDNA. After second-strand synthesis, double-stranded cDNA was used in an *in vitro* transcription (IVT) reaction to generate cRNA (MEGAscript T7 kit; Ambion, Austin, TX). 600 ng of the cRNA obtained was used for a second round of cDNA and cRNA synthesis, resulting in biotinylated cRNA (GeneChip Expression 3’-Amplification Reagents for IVT-Labeling; Affymetrix). Size distribution of the cRNA and fragmented cRNA, respectively, was assessed using an Agilent 2100 Bioanalyzer with a RNA 6000 Nano Assay.

15 μg of fragmented cRNA was used in a 300-μl hybridization containing added hybridization controls. 200 μl of mixture was hybridized on arrays for 16 h at 45°C. Standard post hybridization wash and double-stain protocols (EukGE-WS2v5_450) were used on an Affymetrix GeneChip Fluidics Station 450. Arrays were scanned on an Affymetrix GeneChip scanner 3000.

### GeneChip data analysis

Scanned arrays were first analyzed with Affymetrix GCOS 1.4 software to obtain Absent/Present calls using the MAS5 detection algorithm. Based on a non-parametric statistical test (Wilcoxon signed rank test) it determines whether significantly more perfect matches show more hybridization signal than their corresponding mismatches, leading to a detection call (Absent (A), Present (P) or Marginal (M)) for each probe set [75]. Transcripts were considered as expressed, if their detection call was “Present” in at least one of the two replicates. Subsequently the 16 arrays used in this study (root hairs; [5],[46]) were analyzed with dChip 2006 (https://sites.google.com/site/dchipsoft/) as described in [5] with the only difference that no filter for high variation within the replicates was applied. Annotations were obtained from the NetAffx database (www.affymetrix.com) as of July 2007. The raw data is available at Gene Expression Omnibus under the series number GSE38486 (http://www.ncbi.nlm.nih.gov/geo/query/acc.cgi?acc=GSE38486).

CEL files containing raw expression data of single cell types from roots [6],[47] were obtained from the AREX database (www.arexdb.org) and detection calls analyzed as described above.

Expression data obtained with dChip were imported into Partek Genomics Suite 6.07 for 3D principal component analysis and hierarchical clustering. For the latter Pearson’s dissimilarity was used to calculate row dissimilarity and Ward’s method for row clustering. Additional CEL files from [33] were combined with CEL files in this study, analysed with dChip and expression values imported into Chipster 2.12 [76]. Results of PCA analysis were visualized as scatter plots using Origin 9.

Functional annotation tools of DAVID [77] were employed for enrichment analysis of Gene Ontology (GO) terms (biological process; GO level 5) with the following thresholds: Count ≥2; EASE (modified Fisher Exact P-value) ≤0.05; Benjamini-Hochberg ≤0.05, False Discovery Rate ≤10%. Subsequently genes comprising enriched GO terms were subjected to functional annotation clustering followed by manual analysis to identify GO terms with gene lists showing more than 50% overlaps. For GO terms, for which such high redundancy was identified, only the most representative GO terms were retained.

### Promoter analysis

In order to enhance effectiveness for motif finding, we have delimitated the promoters of apical growth selective genes to -1,000 bp upstream of start codon or predicted transcriptional start sites (TSS), and downstream of adjacent genes if the intergenic regions were less than 1,000 bp. Sequences were obtained from Athena database [78], and predicted TSSs from PlantPromoterDB (ppdb) [79]. Promoter sequences were analyzed by MUSA [51] and Promzea [52], using default values for each parameter. MUSA’s output has shown the distribution of motifs detected through each uploaded sequence (Quorum), ranked by p-value. Detected sequences were queried against PLACE database [53] to find correspondence with previously reported elements. Promzea’s output was compared to known promoter motif databases using STAMP [54].

## Competing interests

The authors declare that they have no competing interests.

## Authors' contributions

JB, ST and FS did experiments and performed the bioinformatic analysis of the data. JB, LD and JF conceived the project, participated in the design of the study and wrote the manuscript. All authors read, corrected and approved the final manuscript.

## Additional files

## Supplementary Material

Additional file 1: Figure S1. Principal component analysis of Arabidopsis transcriptome data. Expression values for 22.800 genes are projected onto the first two principal components (PC1 and PC2). **(A)** The same 8 tissue/cell types as in Figure 1, showing a clear separation of pollen and root hairs from the other tissues. **(B)** Samples as in a, but adding root cell types from Dinneny et al. [33]: A, columella root cap; B, cortex; C, endodermis and quiescent center; D, epidermis and lateral root cap; E, protophloem; F, stele. Click here for file

Additional file 2: Table S1. Data underlying PCAs in Additional file 1: Figure S1, including variance and loadings. Click here for file

Additional file 3: Table S2. List of 1814 genes showing enriched expression in root hairs and its overlap with “root hair genes” or pollen tube up-regulated genes as defined in other studies. Click here for file

Additional file 4: Table S3. Detailed expression date for 4989 transcripts expressed both in root hairs and pollen. In addition information is provided whether a gene is selectively expressed in growing cells, enriched, or depleted. The average fold change (FC*) is given as the lower confidence bound fold change of all relevant comparisons. Transcript that are selectively expressed based on our data set, but not if compared with publicly available ATH1 datasets are denoted with “(X)^#^”. Click here for file

Additional file 5: Table S4. Genes used for comparative Gene Ontology analysis, sorted in their respective functional classes. Click here for file

Additional file 6: Table S5. Selectively expressed and enriched genes in apical growing cells. The first column shows the functional classification of the gene (see also Figure 7). The second column depicts the Affymetrix probe set, followed by TAIR locus (AGI ID) assigned to this probe set and gene annotation in the third and fourth column. In columns five and six the expression values for pollen and root hairs, respectively, are given. The following three columns (7 to 8) depict, if a gene is selectively and /or enriched expressed in root hairs and pollen, followed by the average of the lower confidence bound of the fold change (FC) for apical growing cells. The last two columns give the average FC of pollen and root hairs, respectively. Click here for file

Additional file 7: Table S6. Primer sequences used for RT-PCR on root hair samples and for confirmation of selectively expressed apical growth transcripts. Click here for file
